# Allotype‐Dependent Responses to the Vaccine Candidate Thrombospondin‐Like Protein of *Dictyocaulus viviparus in Calves*

**DOI:** 10.1111/pim.70013

**Published:** 2025-07-16

**Authors:** Frans N. J. Kooyman, Karlijn L. J. Moonen, Rolf Nijsse, Jaap A. Wagenaar, Harm W. Ploeger

**Affiliations:** ^1^ Division of Infectious Diseases and Immunology, Faculty of Veterinary Medicine Utrecht University Utrecht the Netherlands; ^2^ Veterinary Practice “De Keizer” Kessel the Netherlands

## Abstract

The nematode *Dictyocaulus viviparus* causes parasitic bronchitis in cattle. There is a vaccine based on irradiated larvae against this parasite. However, no memory response is induced, and donor calves are needed for culturing the larvae. Therefore, a well‐defined subunit vaccine would be welcomed. Because thrombospondin‐like protein (TLP) is an immunodominant protein from the brush border of adult parasites, it was tested as a vaccine candidate. Calves (*n* = 7) were vaccinated twice with TLP in Quil‐A, and antibody responses, IgG2 allotypes and protection were determined. Protection is defined here as decreased worm counts from challenge infection compared with age‐matched control calves (*N* = 7). Only 27% protection (not significant) was found in the vaccinated calves. However, strong IgG and IgE booster responses occurred after challenge infection, mostly directed against the glycan‐phosphorylcholine moiety of the protein. Most interesting was the difference in protection in calves of the different IgG2 allotypes. The two best protected calves from the vaccinated group were the only two calves of the homozygote IgG2^b^ genotype. Because the IgG2^b^ has a more rigid hinge region than the IgG2^a^ allotype, it is more resistant to parasite proteases or parasite Ig binding proteins, and it is a better complement activator. Therefore, even with the small number of calves from this study, results suggest calves of the homozygous IgG2^b^ genotype might be better protected against *D. viviparus* than calves of other genotypes.

## 1 Introduction

The nematode *Dictyocaulus viviparus* causes parasitic bronchitis in cattle. Symptoms in calves range between a mild to severe respiratory symptoms, weight loss and sporadically, death. In susceptible lactating cows, a reduction in milk yield occurs [1]. Historically, it was assumed only calves in their first grazing season were clinically affected. Older animals were thought to have acquired strong immunity against *D. viviparus*. However, currently, dictyocaulosis is considered a relevant clinical problem in adult dairy cattle too, possibly due to limited exposure [1], leading to decreased animal welfare and economic challenges. Holzhauer et al. (2011) estimated the costs of a lungworm outbreak in The Netherlands at about 160 euro per cow [2].

Prevention against dictyocaulosis is based on a combination of anthelmintic treatment, pasture management and vaccination with irradiated larvae. There are several downsides concerning these methods of prophylaxis. The resistance of nematodes in general against anthelmintics is growing, and research suggests the development of resistance of *D. viviparus* against macrocyclic lactones [3, 4]. Strategic grazing, as used to control infections with gastrointestinal nematodes to prevent lungworm disease, is not practical because of the different epidemiology of *D. viviparus* compared to gastrointestinal helminths. The combined use of anthelminthics with strategic grazing may limit the exposure of calves to *D. viviparus* during their first grazing season, leading to a suboptimal immunologic response in the next grazing season. There is a commercially available vaccine against lungworm (Huskvac, MSD), consisting of two doses of 1000 irradiated L3 larvae that are administered at a 4‐week interval. These attenuated larvae survive for approximately 2 weeks in the host. In this period, the larvae do elicit an immune response, but they die before they can cause disease. Four weeks after the second vaccine dose, calves are protected against infection. Unfortunately, Huskvac possesses some drawbacks. First, the vaccine does not induce long‐lasting immunity without a natural booster infection with *D. viviparus* [5]. In other words, the vaccine induces a poor memory response. Second, infected donor calves are needed for the production of the Huskvac vaccine. Besides these two negative aspects, the composition of Huskvac is undefined, and the nature of the vaccine antigens that provide protection against infection is still unknown.

In search of a new defined vaccine against lungworm disease, a high molecular weight glycoprotein was found as a candidate vaccine antigen. The IgG and IgE responses against this protein were correlated with protection [6]. The high molecular weight glycoprotein, called provisionally GP300, consists of seven thrombospondin domains with a ‘papilin cassette’ and six highly allergenic Kunitz domains and was later identified as thrombospondin‐like protein (TLP) [7]. Figure 3 of that study showed the domains and putative N‐glycosylation sites of TLP of *D. viviparus*. The protein was found in L3 larvae, in the excretory/secretory (E/S) products as well as in the brush border of the gut of the adult lungworm. The protein is N‐glycosylated with one or more phosphorylcholine (PC) groups attached to the N‐glycan moiety and most of the immunological reactivity was against the glycan and/or the PC part [8].

Antibodies against the PC part of this glycoprotein, cross‐react with the PC part of the proinflammatory Platelet‐Activating Factor (PAF). This may cause neutralisation of PAF, leading to less macrophage and eosinophil activation, less chemotaxis and less IgG2 production (down regulation of the inflammatory reaction) [8]. This could conduct a shift away from the proinflammatory response toward a T helper2 response. Less symptoms of illness for the host may be a result of this process. The idea of an immunomodulatory function of PC is not new. Houston et al. (2000) reported about ES‐62, a PC‐substituted glycoprotein of the rodent filarial nematode *Acanthocheilonema viteae*, with the ability to decrease IgG2 responses [9]. ES‐62 is an aminopeptidase and therefore not related to TLP [10]. This might be of less importance, because the immunomodulatory effects were not caused by the protein backbone, but by PC. Similar effects of ES‐62 were obtained with PC attached to bovine serum albumin [9].

Therefore, a vaccination experiment with TLP was conducted. Hypothetically, two possible effects of a TLP vaccine can occur: (1) Vaccination leading to an immune response (IgG, IgE), protecting the calves against a challenge infection or (2) vaccination leading to a decreased inflammatory response (by its PC moiety) without protection against challenge infection, but with decreased inflammation and clinical signs.

## 2 Materials and Methods

### 2.1 Calves and Parasites

Fourteen male Holstein‐Friesian calves (4 months old at Day 0) were housed indoors. The calves had *ad libitum* access to fresh water and hay. They were fed a mixture of maize and concentrate sufficient for growth and maintenance.

The calves were randomly assigned to a vaccine (*n* = 7) and a control group (*n* = 7). The group size was estimated from earlier experiments, where very low primary doses induced significant protection against challenge infection [11]. On Days 0 and 21, the vaccine group received 100 μg of purified TLP and 750 μg Quil‐A in 2 mL intramuscularly. The control group received only 750 μg Quil‐A. Subsequently, on Day 42, both groups were orally infected with 500 L3 per calf. On Day 72, all calves were slaughtered. In order to determine protection, worms in the lungs of the slaughtered calves were counted. The worms were collected by means of the modified Inderbitzin method, as described [12], with some minor modifications. Each pair of lungs was washed with 10 L of saline containing 2 mM EDTA instead of water to prevent collapse of the worms. A tube attached to a pump was inserted in the arteria pulmonalis. By applying moderate pressure (10 L of saline in 15 min) the alveoli collapsed and the worms were washed with the fluid out of the trachea. Worms were collected on a 150 μm sieve. The rinsed lungs were chopped into pieces and were subjected to a modified Baermann method to collect as many worms as possible. This modified Baermann method followed [12] with a minor modification: bucket contents were sieved after 4 h instead of overnight. All collected worms were preserved in 70% alcohol to be sexed and counted.

### 2.2 L3 Larvae for Challenge Infection

L3 larvae used for challenge infection were obtained from faecal cultures from two donor calves from the Faculty of Veterinary Medicine, Utrecht University. The faecal cultures were kept 15°C for 1 week. Subsequently, by submersing the faeces in water, the L3 larvae were collected. Larvae were approximately 3 months old when used. Viability of the L3 larvae was confirmed by Bile Agar Migration test [13].

### 2.3 Clinical Observations

Before any handling of the calves took place, respiration rate and coughing score were determined three times a week, from Day 35 onward. Respiration rate and coughing score were always recorded between 12.30 and 13.00 pm, without fixation of the calves. Respiration rate was determined by counting breaths for half a minute and expressed as breaths/min. Coughing was evaluated on frequency (no, occasionally, regularly, frequently) All evaluations were performed by the same trained veterinary student.

### 2.4 Faecal Samples

On Day 0 and from Day 59 onward, rectal faecal samples were collected from each calf. Faecal larval counts were carried out at Days 0, 59, 63, 64, 65, 66, 67, 70 and 71 by means of the Baermann method using 30 g of faeces. Results were expressed as larvae per gram of faeces (LPG).

### 2.5 Collection of Blood Serum

From 1 week before the study began, blood samples were collected weekly (for IgG ELISA) or biweekly (for IgE ELISA) from the vena jugularis. The serum was collected and stored at–20°C until testing.

### 2.6 Purification of TLP

Purification of TLP was performed as described [8], up to the point of the precipitation with trichloroacetic acid and acetone. In this publication, the provisional name GP300 was used for TLP. The purification was checked by SDS‐PAGE and silver stain, and protein content was determined with Bradford (ThermoFisher Scientific).

### 2.7 Deglycolysation of TLP

Deglycosylation of TLP was performed as described [14]. TLP was deglycosylated by incubating a mixture of 100 μL TLP (3 μg/μL), 100 μL 1% SDS, 120 μL H_2_O, 25 μL 10% 2‐mercaptoethanol and 50 μL deglycosylation buffer for 5 min at 100°C. After cooling to room temperature (RT), 100 μL 10% NP‐40 and 5 μL PNGase F (peptide‐N‐glycosidase, Roche cat nr. 1,365,177) were added. This final mixture was incubated overnight at 37°C. Incubation was stopped by heating for 3 min at 100°C. Mock treated samples (all except the PNGase F) were incubated simultaneously.

### 2.8 SDS‐PAGE

Purification and deglycolysation of TLP was verified by SDS‐PAGE (7.5% gel). Antigen (0.1 μg) was loaded into each lane under reduced conditions and gels were silver stained. Verified mock treated and PNGase F treated TLP fractions were used for ELISA.

### 2.9 Serology

#### 2.9.1 Enzyme Linked Immunosorbent Assay (ELISA'S)

For all ELISA's, Greiner Bio One high binding plates were coated overnight at RT with antigen in 0.06 M Na_2_CO_3_, pH 9.6. Dilutions of sera and antisera were made in Phosphate Buffered Saline (PBS) containing 0.1% gelatine and 0.05% Tween‐20 (PBS‐GT). The utilised ELISA reader was a Ceres UV 900 C.

#### 2.9.2 IgG2 ELISA'S

##### 2.9.2.1 TLP‐Specific IgG2 ELISA

Plates were coated with TLP/Mock treated or TLP/PNGase F treated (0.2 μg/mL). The test sera were diluted 1:2000. Mouse anti‐IgG2 specific monoclonal antibody (Bio‐Rad, mca 626, 1:500) was applied, followed by 1:2000 goat antimouse Ig/AP (DAKO cat nr. D0486). P‐nitrophenyl phosphate (PNPP, Pierce, cat nr. 37620ZZ) was used as a substrate and the plates were incubated for 30 min at RT and read at 405 nm.

##### 2.9.2.2 TLP‐Specific IgG1 ELISA

The same protocol as described for IgG2 ELISA, with the only exception that the IgG1 isotype specific monoclonal antibody (Bio‐Rad, mca 627, 1:1000) was used.

##### 2.9.2.3 Total IgG2 ELISA'S

Total IgG2 was measured in two ways, with a secondary polyclonal antibody or monoclonal antibody (mca 626). Total IgG2 with the polyclonal antibody was performed with the Bovine IgG2 ELISA quantification kit (Bethyl laboratories.inc.). Coating antibodies were used in a 1:100 dilution, sample sera were used in 1:25,000, and sheep anti‐Bovine IgG2/HPO conjugate was used in a 1:10,000 dilution with TMB as substrate (10 min). Development was stopped by the addition of 100 μL/well 2 M H_2_SO_4_, and plates were read at 450 nm. Total IgG2 ELISA with the monoclonal antibody was performed with the Bovine IgG2 ELISA quantification kit (Bethyl laboratories.inc.), but instead of the polyclonal conjugate, mouse anti‐IgG2 specific monoclonal antibody (Bio‐Rad, mca 626) was used (1:2000), followed by a 1:2000 goat antimouse Ig/AP (DAKO cat nr. D0486). PNPP was used as a substrate, and the plates were read after 60 min at RT at 405 nm.

##### 2.9.2.4 Total IgE ELISA

ELISA was based on [6]. Plates were coated with purified monoclonal antisheep IgE (IE7), also recognising bovine IgE, in coating buffer at 3 μg/mL. Sera were heat treated before use (1 h at 56°C) and subsequently, the sera were diluted 1:5. Standard sera were heat treated in the same way as the test sera. The standard sera were diluted as follows: 1:5, 1:10, 1:20, 1:100 and 1:200, and were administered to the plates in duplicates. Polyclonal rabbit antibovine IgE 1:1000 was utilised as second antibody solution. Next, plates were incubated with 1:4000 goat antirabbit Ig/AP conjugate (DAKO, cat nr. D 0487). PNPP was used as substrate. The plates were read after half an hour incubation at RT at 405 nm. Obtained ODs were expressed in mU of standard serum (1000 mU = undiluted standard serum).

#### 2.9.3 Allotyping of IgG2 From Serum

The PCR for IgG2 allotypes of the calves were performed after necropsy, when serum was the only available source for template DNA. Serum (700 μL) was spun for 20 min at 20,000 g. The pellet was digested in 100 μL wormlysis buffer/protein K (WLB/Prot K) [15] for 3 h at 56°C, followed by 15 min at 95°C. Nested PCR was performed with F1‐IGG2 (5′ CGG GGT GCT GTG AAC CA 3′) and R1‐IGG2 (5′ TGT CTT TGG GTT TCG GTG G 3′) in the first round and F2‐IGG2 (5′AGT GTG TCA GGG GAG GC 3′) and R2‐IGG2 (5′ GTT CCC TCA CGC CTA ATG G 3′) in the second round. The sequences of the F1‐IGG2 and R2‐IGG2 primers were given by [16] and enabled the specific amplification of IgG2. The F2‐IGG2 and R1‐IGG2 primers were specifically designed for nested PCR, required due to the low template concentration in serum. The conditions for first and second round were the same: 98°C (30 s) once and 40 cycles of 98°C (10 s), 56°C (30 s) and 72°C (60 s), followed by 72°C (7 min) once. Phusion hostart II High‐Fidelity DNA polymerase (Thermofisher, F‐549S) was used for all PCR reactions. Amplification products were validated by agarose gel electrophoresis, purified by GFX PCR DNA and GelBand Purification Kit (Cytiva, UK) and sent to Baseclear (Leiden, The Netherlands) for Sanger sequencing. Analyses of the DNA sequences were performed in DNASTAR Lasergene 17 and MEGA 11. Based on the hinge region, the IgG2 fragments were identified as either IgG2^a^, IgG2^b^ or mixed IgG2^a/b^allotypes, formerly known as IgG2(A1), IgG2(A2) and IgG2 (A1/A2) allotypes, respectively [16].

### 2.10 Statistical Analysis

Protection against infection is defined as: resistance against a primary infection with *D. viviparus* in vaccinated animals compared to an age‐matched nonvaccinated control group based on the worm counts. Statistical analysis was carried out using SPSS software package (version 29.0.2.0). Nonnormally distributed data were analysed using a Mann–Whitney U test for group comparisons and Spearman Rank correlation for associations. Correlations and differences were considered significant when *p* ≤ 0.05. Unless otherwise indicated, no Bonferroni corrections were applied.

## 3 Results

### 3.1 Purification and Deglycosylation of TLP

**FIGURE 1:**
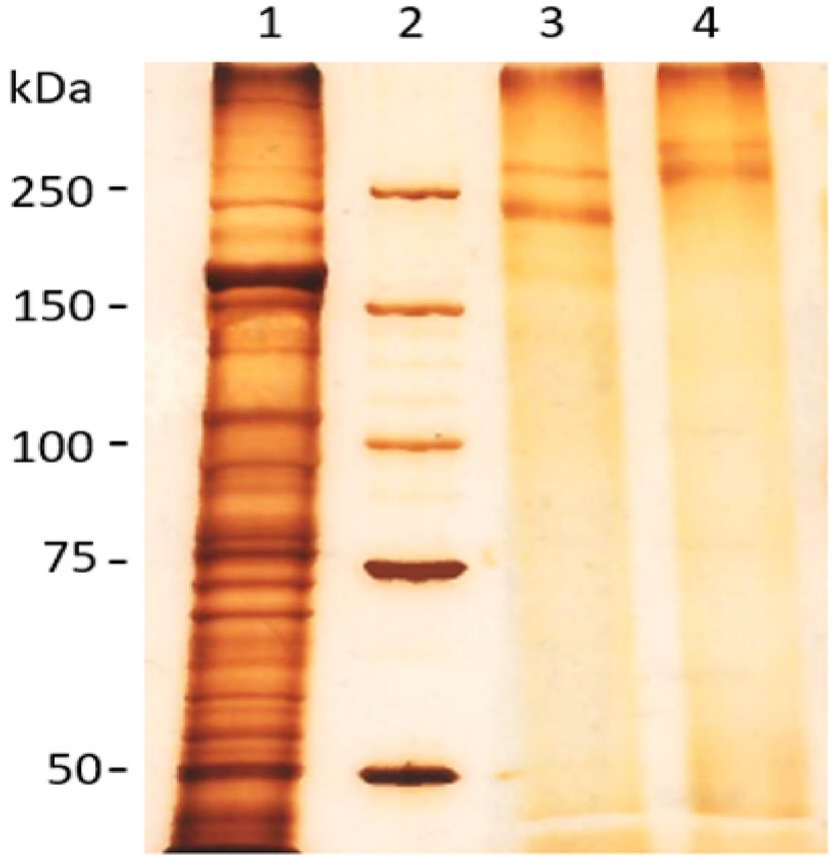
SDS‐PAGE with silver staining. Crude worm extract (1) starting material for the purification of TLP. TLP after PNGase F treatment (3) or after mock treatment (4). Size of the molecular weight markers (2) is indicated.

The purification of TLP used in the vaccine, was compared with a crude worm extract (Figure 1). The two bands, characteristic for TLP, are visible without contamination and the bands in the PNGase F deglycosylated fraction are of a lower molecular weight than those of the mock treated fraction, confirming the loss of the glycan moiety.

### 3.2 Calves, Worm Counts and Larval Excretion

The postmortem worm counts of the calves are given in Table 1. In order to link the individual calves with their worm counts, the calves will be indicated through the paper by their individual worm counts. The number of recovered worms was 26.9% less in the vaccinated group than in the control group, but this difference was not significant.

**TABLE 1:** Total worm counts of individual calves of the vaccinated (*n* = 7) and control group (*n* = 7) after necropsy. The individual calves are identified by their worm counts.

	Vaccinated	Control
	9	18
	11	61
	49	64
	52	86
	68	97
	113	120
	127	141
	61.28	83.86
Reduction	**27%**	**—**

**FIGURE 2:**
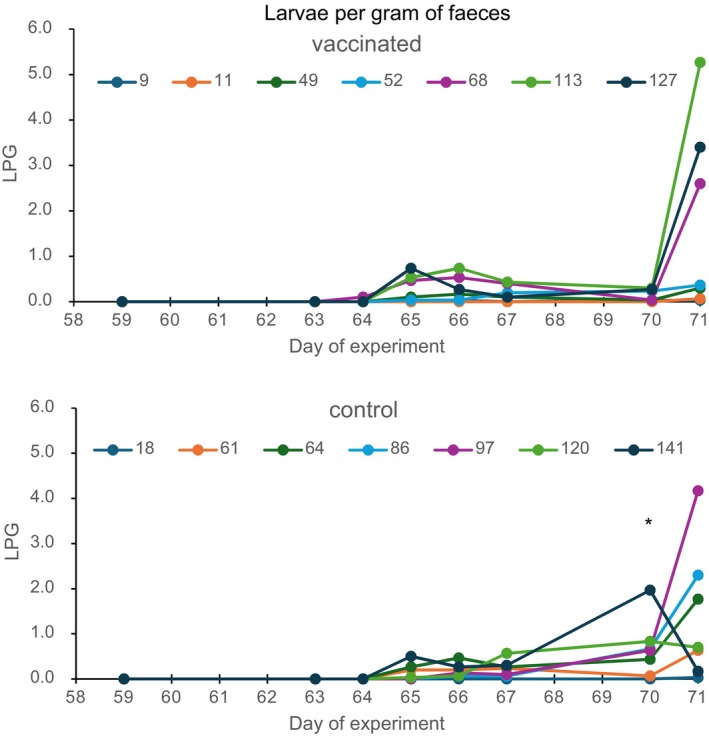
Larvae per gram of faeces (LPG) of the vaccinated and control calves. Challenge infection of 500 larvae was given on Day 42. Calves are indicated by their worm counts. * significant (*p* = 0.5) higher in the control group.

De larval excretion of the individual calves is given in Figure 2 and Table S1. No significant differences in total excreted larvae were found between the vaccinated and the control groups. The LPG of the vaccinated calves was only at Day 70 significantly (*p* = 0.05) lower than of the control calves. The worm counts were significantly and positively correlated (*p* < 0.01) with the total larval excretion (not shown).

### 3.3 Clinical Signs

#### 3.3.1 Coughing and Respiration Frequency

Coughing score between the vaccinated and control group was not different, except on Day 63, where coughing score was significantly higher (*p* = 0.04) in the control calves (Figure S1). Respiration frequency (breaths/min ± std.dev.) was in general high in vaccinated calves (56.0 ± 13), as well as in control calves (60.4 ± 12). Only on Day 58 was the respiratory rate significantly higher (*p* = 0.05) in the control calves (Figure S2).

### 3.4 Antibody Responses

#### 3.4.1 Total IgE Levels

**FIGURE 3:**
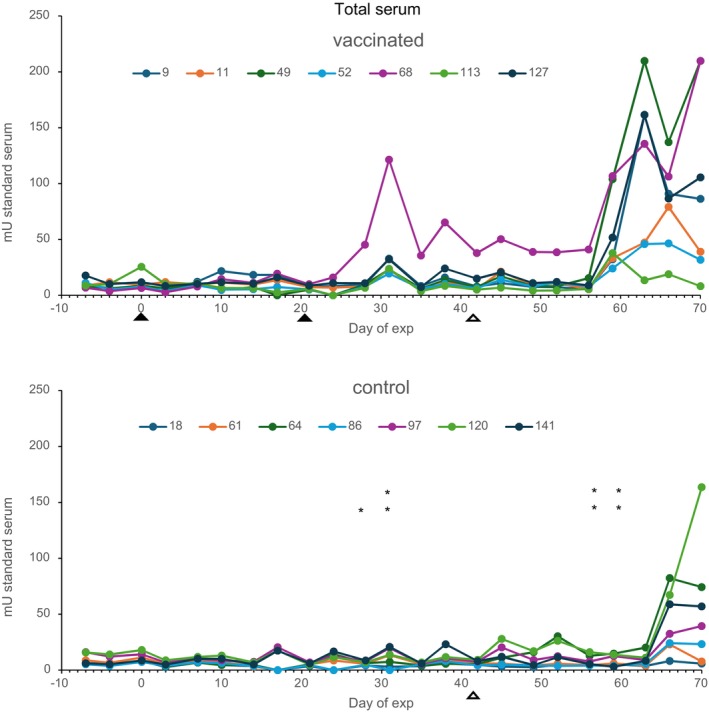
Total serum IgE in vaccinated and control calves. Filled arrowheads indicate the time of vaccinations and open arrowheads indicate the time of challenge infection. Significantly higher total IgE in vaccinated calves compared with control calves *p < 0.05, **p < 0.005.

Total IgE levels in serum were measured in the vaccinated and control calves (Figure 3). No effect after the first vaccination was seen, but a significant increase in total IgE levels was seen 7 and 10 days after the second vaccination (Days 28 and 31 of experiment). After that, levels decreased slightly but rose again in the vaccinated group and were significantly higher than in the control group 17–21 days after challenge infection (Days 59 and 63 of the experiment). At the end of the experiment, total IgE levels were elevated in both groups. No correlations between total IgE and worm counts were found.

#### 3.4.2 IgG Responses

**FIGURE 4:**
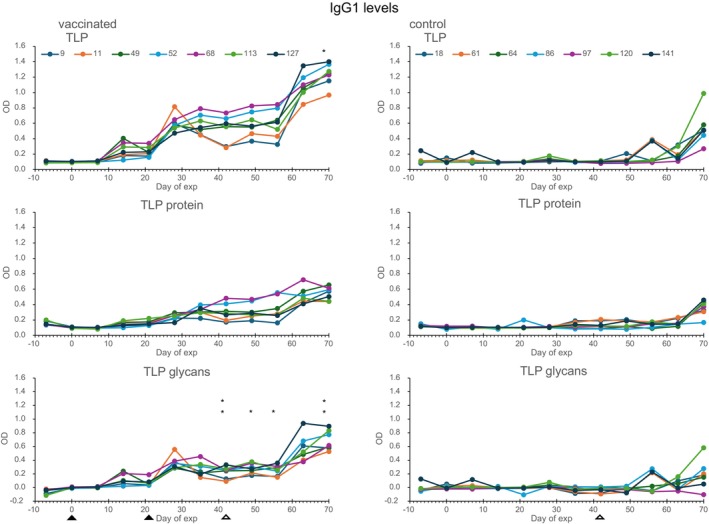
IgG1 responses of the vaccinated calves (left column) and the control calves (right column) against TLP (upper panel), TLP protein moiety (middle panel) and TLP glycan moiety (TLP minus TLP protein, lower panel). Filled arrow heads indicate time of vaccination and open arrow heads indicate day of challenge infection. OD correlates with worm counts *p < 0.05, **p < 0.005.

**FIGURE 5:**
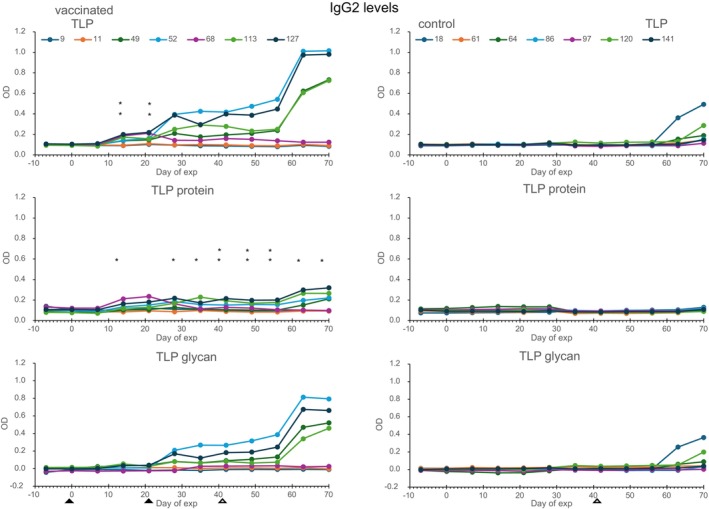
IgG2 responses measured with mab of the vaccinated calves (left column) and the control calves (right column) against TLP (upper panel), TLP protein moiety (middle panel) and TLP glycan moiety (TLP minus TLP protein, lower panel). Filled arrow heads indicate time of vaccination and open arrow heads indicate day of challenge infection. OD correlates with worm counts *p < 0.05, **p < 0.005.

IgG responses against intact TLP, deglycosylated TLP (TLP protein) and against intact TLP minus the deglycosylated TLP (TLP glycans) were measured (Figures 4 and 5). IgG1 responses against TLP were found 14 days after the first vaccination, and a booster response was found after the second vaccination and after challenge infection. A marked rise in IgG1 levels occurred 7 days postsecond vaccination, followed by a sharp decline in calves 9 and 11, which also exhibited the lowest worm counts at necropsy. This response was predominantly directed against the TLP glycans. The TLP specific IgG1 levels at Day 70 and the TLP glycan specific IgG1 levels at several days between Day 42 and 70 were significantly positively correlated with worm counts in the vaccinated calves (see Figure 4). The control group showed only a low response and only after challenge infection.

TLP specific IgG2 responses measured with monoclonal antibodies (mab) are shown in Figure 5. Already 14 days after the first vaccination a response was found, and a booster response was found after the second vaccination and challenge infection. Interestingly, in the two calves with the lowest worm counts (calves 9 and 11) no IgG2 response was detected. Deglycosylation of TLP (TLP protein) removed almost all IgG2 binding in all calves. The TLP and TLP‐protein specific IgG2 levels in the vaccinated group were significantly positively correlated with worm counts on many days from Day 14 onward, while IgG2 responses in control calves were low, even after infection.

**FIGURE 6:**
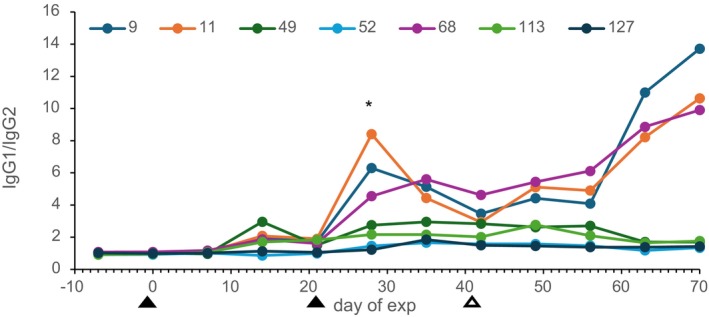
The ratio of IgG1/IgG2 response against TLP in the vaccinated calves. Filled arrow heads indicate the vaccinations and open arrow head indicates the time of challenge infection. OD correlates negatively with wormcounts *p < 0.05.

The sharp peak in IgG1 response against TLP and unresponsiveness in IgG2 response of calves 9 and 11 of the vaccinated group was remarkable. Therefore, the ratio of IgG1/IgG2 response against TLP was calculated (Figure 6). Calves 9 and 11 had the highest IgG1/IgG2 ratio, while most of the calves with the higher counts remained at a ratio of about 1. The ratio at Day 28 (7 days after the second vaccination) was positively correlated with protection (*p* = 0.04).

Total IgG2 ELISA's with mab as well as with polyclonal antibodies (pab) anti‐IgG2 were performed to test whether the calves 9 and 11 are deprived of IgG2 or are having an IgG2 allotype not recognisable by the mab, because mab might be selective for specific allotypes of IgG2.

#### 3.4.3 Total IgG2 ELISA

**FIGURE 7:**
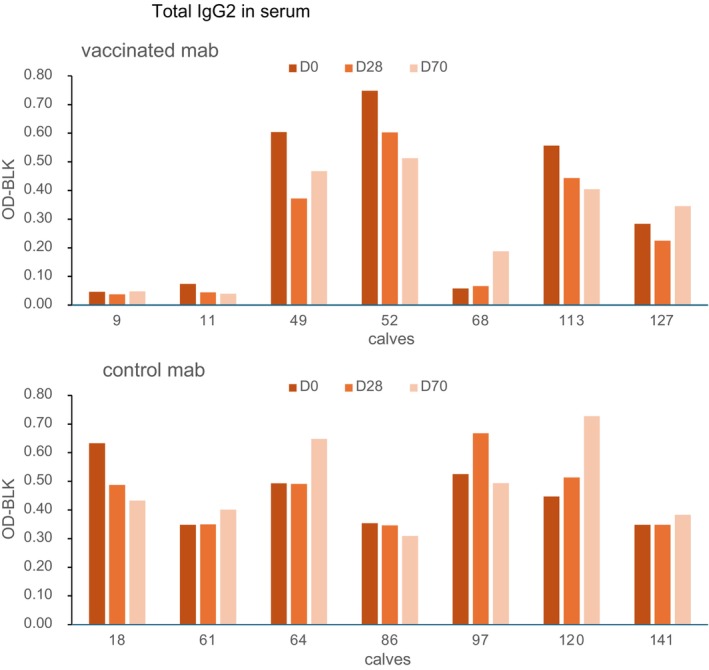
Total IgG2 in serum of Day 0, 28 and 70 from vaccinated (top) and control (bottom) calves. Measured with mab directed against bovine IgG2.

Total IgG2 ELISA's with pab against bovine IgG2 were performed on sera from the vaccinated calves from Day 0, 28 and 70. All calves showed similar IgG2 levels and some increase or decrease over time (Figure S3). When total IgG2 ELISA's were performed with the same mab against bovine IgG2 used in the TLP specific ELISA (Figure 7), the IgG2 levels in the vaccinated calves varied widely. The calves with very low outcomes in mab TLP ELISA (calves 9 and 11) showed comparable outcomes here. These calves, with IgG2 recognised by the pab, but not by the mab anti‐IgG2, had the lowest worm counts. For confirmation, the IgG2 allotype of the vaccinated calves was determined by PCR and sequencing.

#### 3.4.4 IgG2 Allotypes in Vaccinated Calves

Sera, as the only available source, from the vaccinated calves were used as templates for amplification and sequencing of the IgG2 encoding fragment. Based on the hinge region, the calves were identified as homozygote IgG2^a^, homozygote IgG2^b^or heterozygote IgG2^a/b^ (Table S2). The deduced amino acid sequence resulted in four heterogeneous loci in the hinge region between the IgG2^a^ and IgG2^b^ allotype (Table 2). The genotypes correspond with the mab binding in the total IgG2 assay; the homozygote IgG2^b^ showed no binding to mab (calves 9 and 11), the homozygote IgG2^a^ showed the highest mab binding (calves 49, 52 and 113) and the heterozygote IgG2^a/b^ had intermediate mab binding (calves 68 and 127).

**TABLE 2:** Deduced amino acid sequences of the hinge region of IgG2 allotypes from the vaccinated calves. Given amino acids correspond to the deduced amino acids 246–260 of ref_IgG2^a^.

	Sample	Allotypes					Amino acid sequences										
ref_IgG2^a^,^a^	X16702.1	2^a^	V	S	S	D	C	S	K	P	N	N	Q	H	C	K	S
ref_IgG2^b^,^a^		2^b^			I					C	H			P			
Calf	9	2^b^			I					C	H			P			
Calf	11	2^b^			I					C	H			P			
Calf	49	2^a^															
Calf	52	2^a^															
Calf	68	2^a^/2^b^			I/S					C/P	H/N			P/H			
Calf	113	2^a^															
Calf	127	2^a^/2^b^			I/S					C/P	H/N			P/H			

^a^ Carvalho et al. (2011). Reference haplotype (×16702.1) identical to IgG2^a^. Variants identical to IgG2^b^ allotype.

### 3.5 Allotypes and Protection

The two best protected calves from the vaccinated group (calf 9 and 11) were the only calves of the homozygote IgG2^b^ genotype. Within the vaccinated group, the homozygote IgG2^b^ type had significantly lower worm counts (*p* = 0.05) compared to the other genotypes (IgG2^a/a^ and IgG2^a/b^ combined). Based on the results of the total IgG2 ELISA, the control calves can be assumed to be of the IgG2^a^ or IgG2^a/b^ genotype, and when ignoring the effect of vaccination, the homozygote IgG2^b^ calves had significantly lower worm counts (*p* = 0.02) than the other 12 calves.

## 4 Discussion

The TLP vaccine induces only a nonsignificant decrease in worm counts of 27% compared with the control group. Larval excretion was not reduced in the vaccinated group, except at Day 70. Symptoms (coughing and increased respiration) were only mildly diminished around Day 60, about 18 days after the challenge infection, which is the time the first symptoms were expected. This indicates that the effect of the vaccine on protection (hypothesis 1) or reduction of the symptoms by decreased inflammatory responses (hypothesis 2) was limited. Nevertheless, the IgE, IgG1 and IgG2 antibody levels were strongly boosted after the vaccination and after the challenge infection. Only on specific timepoints they were positively correlated with worm counts. The most surprising antibody responses were found in the two best protected calves (calves 9 and 11). They were the only calves of the IgG2^b^ genotype and they showed a short‐lived high peak of IgG1 1 week after the second vaccination, but no IgG2 responses could be detected. Therefore, the IgG1/IgG2 ratio (proxy of the Th2/Th1 ratio) at that timepoint was the only occasion in which a positive correlation was found between protection and antibody response in the vaccinated calves.

TLP contains multiple kunitz domains [7]. It is known that some proteins, because of these repeated kunitz domains can induce IgE responses [17], like for example the kunitz‐type inhibitor in *Anisakis simplex* major allergen (Ani 1) [18]. This suggest that the kunitz domains in TLP are the possible cause for the high total IgE levels in the vaccinated calves. The glycoprotein TLP also contains PC. The possible anti‐inflammatory effect of PC and therefore on reducing the symptoms, is described in literature [19]. Part of this anti‐inflammatory effect is due to the reduction of the IgG2 response by the PC moiety of the parasitic glycoprotein ES‐62. The same effect was achieved with artificially attached PC to BSA [9]. In the present study there were IgG2 responses found in most calves against TLP and most of the responses were directed against the glycan‐PC moiety and not against the protein part of the TLP. Therefore, we did not find inhibition of the IgG2 responses. Although, for a better comparison, also calves should be tested which were vaccinated with deglycosylated TLP. The IgG2 inhibiting effect of PC described in literature was found in mice. Therefore, absence of inhibition of the IgG2 responses may be related to the host species, because the IgG2 isotypes of mice and cattle are, despite their similar names, not orthologous. Immunomodulatory effects of PC present on proteins of *A. viteae* results in decreased IFNγ and decreased inflammation [9]. If the TLP vaccine would have decreased inflammatory responses, it would have resulted in less symptoms. However, this does not seem to be the case, apart from a higher respiratory rate at Day 58 and a higher coughing rate at Day 63 in the control calves.

The glycoprotein TLP is present, among other places, in the brush border of the gut of the adult worm [7]. An ortholog of TLP, also containing PC, is known to be present in an intestinal membrane fraction (H‐gal‐GP) of *Haemonchus contortus*, a parasitic nematode of sheep [7, 20]. H‐gal‐GP, together with H11, forms a complex of many glycoproteins. Immunisation with these native glycoproteins can confer a high level of protection [21]. However, the protective effect depends on the native state of the protein. Solubilisation of the complexes, denaturation of the protein, deglycosylation or recombinant production all resulted in loss of protection upon immunisation [22, 23]. Therefore, the commercial vaccine Barbervax, consisting of H‐gal‐GP and H11, contains the native glycoprotein complex isolated from *H. contortus* . The vaccine must be administered many times, and field infection is needed for booster response. Barbervax is the first commercial subunit vaccine against gastrointestinal nematodes and is a great achievement for veterinary parasitology. Nevertheless, the vaccine works only with native nondenatured proteins, and several applications were required. In light of these difficulties, the low level of protection by TLP in the current study with only two doses is not too surprising. Although the TLP was isolated from *D. viviparus*, it was separated from the extracellular matrix, solubilised and denatured, and it is only a minor ingredient of the intestinal membrane fraction. Nevertheless, the antibody responses were strong, but hardly any protection was found, similar to recombinant H‐gal‐GP in sheep [22].

The most conspicuous results were the putative absence of the IgG2 responses in the two best protected calves from the vaccinated group (calves 9 and 11). Therefore, the IgG2 responses were analysed in the vaccinated calves in more detail. Anti‐IgG2 monoclonals preferentially recognised the IgG2^a^ over the IgG2^b^ allotype [24]. To check whether the absence of IgG2 responses in these calves was caused by the absence of IgG2 or because the mab did not recognise the IgG2 allotypes, total IgG2 levels were measured in ELISA with mab and with pab. With the pab, IgG2 was detected and the IgG2 levels were similar in all vaccinated calves, but with the used mab (mca626) no IgG2 was measured in the two best protected calves. Therefore, the IgG2 allotypes of the vaccinated calves were determined by PCR and sequencing. Indeed, the two best protected calves were of the homozygote IgG2^b^ genotype. The three calves with the highest IgG2 levels were the three homozygote IgG2^a^ calves and the two with intermediate levels were of the IgG2^a/b^ genotype. This confirmed the observations of Butler et al. (1987) about the bias of mab for the IgG2^a^ allotype [24]. Noble et al. (2024) demonstrated recognition of bovine IgG2 by the same mab (mca 626) used by us and that it recognised specifically IgG2 and not IgG1 or IgG3 [25]. However, the only IgG2 variant tested was IgG2^a^ (accession number KT761528.1), the allotype that was also recognised by the same mab in our study. Altogether, this suggests that the calves of the IgG2^b^ genotype are better protected against the challenge infection than calves of the IgG2^a^ or IgG2^a/b^ genotypes, but that the expressed IgG2^b^ antibody was not detected by the mca 626. The only study we could find dealing with IgG2 allotypes and protection against *Dictyocaulus* was from Scott et al. (1996) [26]. They also found IgG2^a^ responses in a calf with high LPG, but no IgG2^a^ response in a calf with low LPG, presumably because it was of the homozygote IgG2^b^ genotype.

Can the putative better protection by IgG2^b^ be explained by differences between IgG2^a^ and IgG2^b^? Certain characteristics of IgG2^a^ and IgG2^b^ point in that direction. The hinge region is practically the only region that differs between the IgG2 allotypes, therefore, most allotype specific mab's are directed against this region. This region is in the IgG2^b^ allotype more rigid [16] and therefore, it is more difficult to raise antibodies against it. For the same reason, IgG2^b^ is also a much stronger complement activator than IgG2^a^ [27], less prone to degradation by proteinases derived from pathogens [28] and less prone to IgG binding proteins of ticks [16]. Noble et al. (2023) could not demonstrate complement activation by bovine IgG2 [29], but that was because only the IgG2^a^ allotype was used and it supports in this way the results of Bastida‐Corcuera et al. (1999) [27]. Complement activation can play a role in protective responses in other helminth infections that reside within the blood or tissues. The fact that many parasites developed strategies to evade complement activation emphasise the importance of it [30]. Passive immunisation against *D. viviparus* can be achieved by serum transfer [31], underlining the importance of antibodies alone or in combination with complement activation. It is therefore likely that complement activation is important in immunity against *D. viviparus*, especially in the early stage when the larvae are small and migrating in the blood and the lymph. However, to the best of our knowledge, no other studies on the effect of IgG2 allotypes and/or complement and susceptibility for *D. viviparus* were conducted. In future studies on responses against vaccine candidates, it is advised to test the specificity of the used anti‐IgG2 monoclonal antibodies for the different IgG2 allotypes and conduct genotyping of the IgG2 allotypes.

## Author Contributions

K.M. performed most of the experiments. R.N., J.W. and H.P. helped with the design of the study. F.K. designed most of the study and performed some of the experiments. All authors contributed to the writing of the manuscript and all authors approved the manuscript, except H.P. who died during the process.

## Disclosure

The authors have nothing to report.

## Ethics Statement

The experiment was approved by the ethical commission of the Utrecht University (number 2010.ii.04.083).

## Peer Review

The peer review history for this article is available at https://www.webofscience.com/api/gateway/wos/peer‐review/10.1111/pim.70013.

## Supporting information


Data S1.

## Data Availability

The data that support the findings of this study are available from the corresponding author upon reasonable request.
