# Supplementary material for: Allotype‐Dependent Responses to the Vaccine Candidate Thrombospondin‐Like Protein of Dictyocaulus viviparus in Calves
Source: Parasite Immunol. 2025 Jul 16;47(7):e70013. doi: 10.1111/pim.70013 (PMC12267107; doi:10.1111/pim.70013)
Supplement: Supplementary file 1 — Data S1. [file PIM-47-e70013-s001.pdf]

Table S1. Larval excretion per gram feces (LPG) of the vaccinated and control calves of day 59 to 71 of the experiment. Challenge infection of 500 larvae was given on day 42.

vaccinated calves

day	9	11	49	52	68	113	127	mean
59	0	0	0	0	0	0	0	0
63	0	0	0	0	0	0	0	0
64	0	0	0	0	0.1	0,00	0,00	0,00
65	0	0	0.10	0.03	0.47	0.53	0.73	0.27
66	0.03	0	0.17	0.03	0.53	0.73	0.26	0.25
67	0	0	0.10	0.20	0.40	0.43	0.10	0.18
70	0.03	0	0.03	0.23	0.03	0.30	0.27	0.13
71	0.03	0.07	0.30	0.37	2.60	5.27	3.40	1.72
Total	**0.10**	**0.07**	**0.7**	**0.87**	**4.13**	**7.27**	**4.77**	**2.56**

control calves

day	18	61	64	86	97	120	141	mean
59	0	0	0	0	0	0	0	0
63	0	0	0	0	0	0	0	0
64	0	0	0	0	0	0	0	0
65	0	0.20	0.27	0	0	0.03	0.5	0.14
66	0	0.20	0.47	0.07	0.13	0.07	0.27	0.17
67	0	0.23	0.27	0.07	0.10	0.57	0.30	0.22
70	0	0.07	0.43	0.67	0.63	0.83	1.97	0.66
71	0.03	0.63	1.77	2.30	4.16	0.70	0.17	1.40
Total	**0.03**	**1.33**	**3.20**	**3.10**	**5.03**	**0.28**	**3.20**	**2.31**

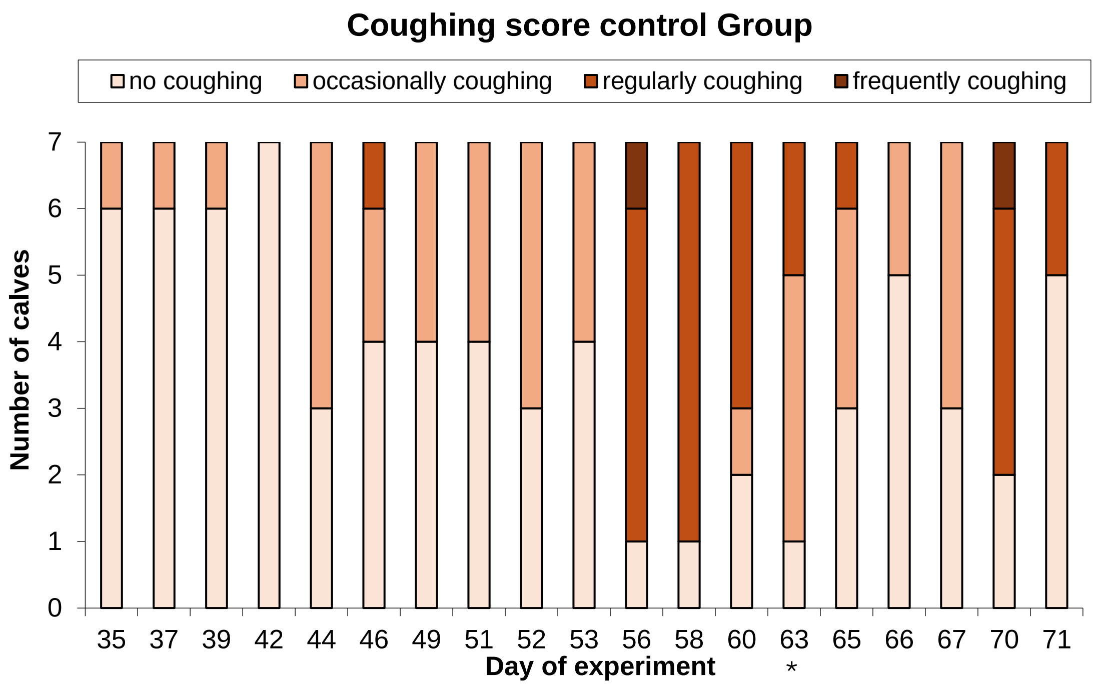

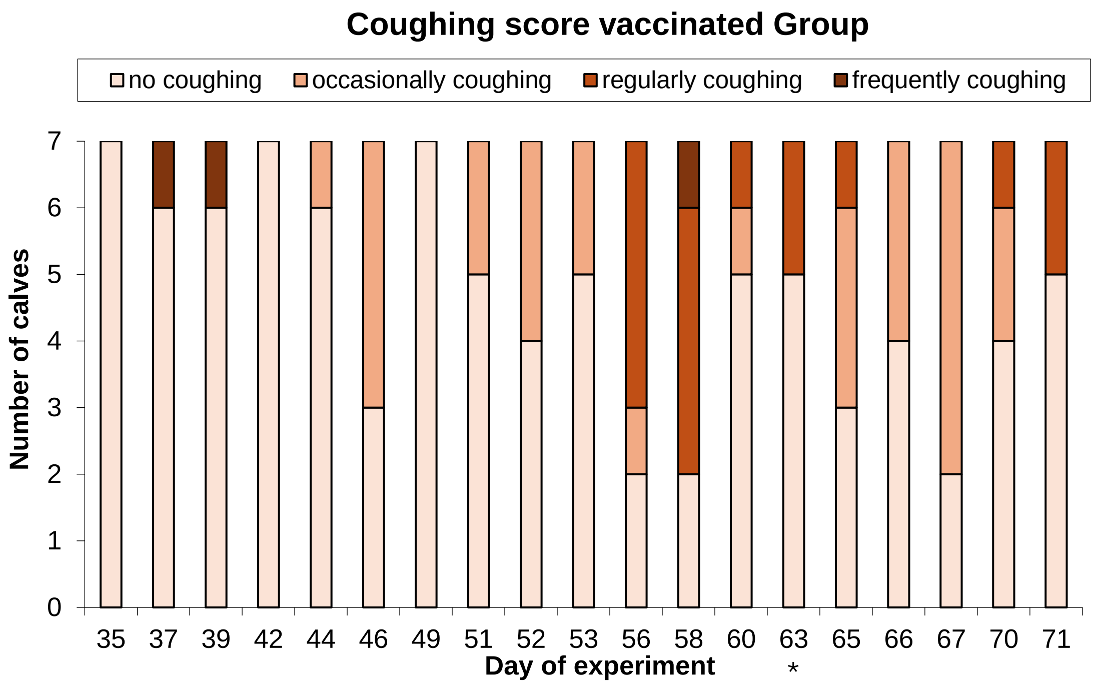

Figure S1. Coughing frequency in control (upper panel) and vaccinated calves (lower panel) no coughing=0, occasionally coughing=1, regular coughing=2 and frequently coughing=3.

* Coughing was significantly (p=0.38) less in the vaccinated calves on day 63.

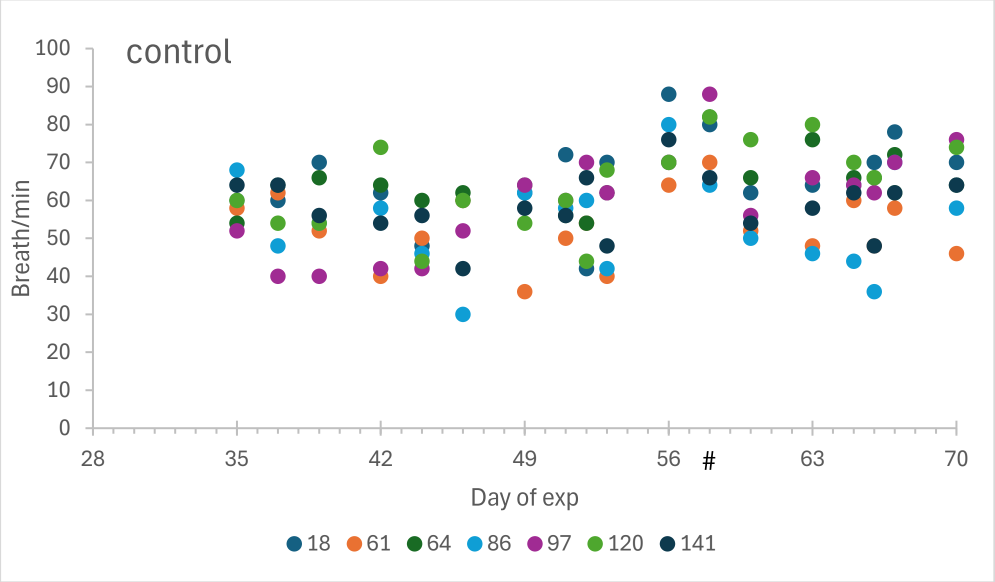

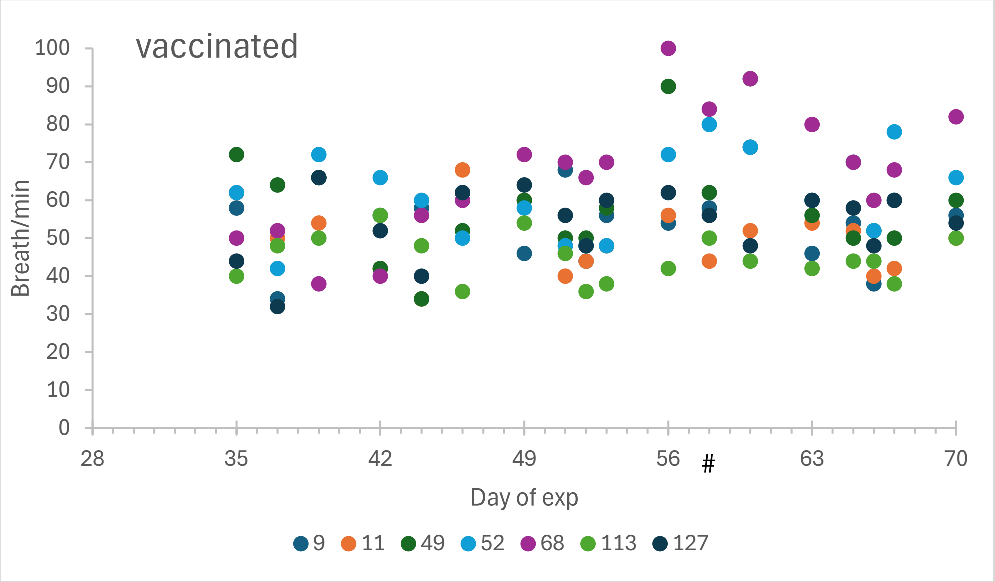

Figure S2. Respiration frequency in control (upper panel) and vaccinated calves (lower panel).
Respiration on day 58 was lower in vaccinated than in control calves (p=0.05).

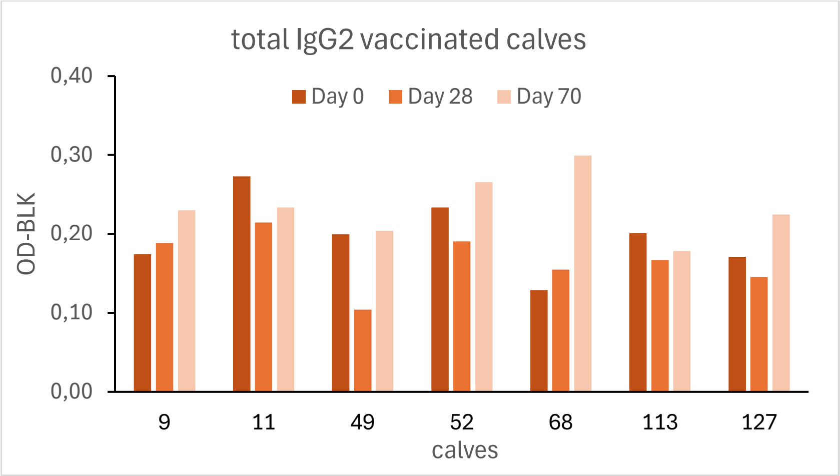

Figure S3. Total IgG2 in serum of day 0, 28 and 70 from vaccinated calves. Measured with sheep anti-bovine IgG2 pab.

Table S2. Nucleotide sequences of the hinge region of IgG2 allotypes from the vaccinated calves and reference sequences. Given nucleotides correspond with nucleotides 746 to 790 of reference sequence IgG2[a]

	sample	*allotype*	**nucleotide sequence**														
*ref_IgG2[a]**	*X16702.1*	*A1*	G T C	T C C	A G T	G A C	T G C	T C C	A A G	C C T	A A T	A A C	C A G	C A T	T G T	A A G	T C G
*ref_IgG2[b]**		*A2*	. . .	. . .	. T .	. . .	. . .	. . .	. . .	T G .	C . .	. . .	. . .	. C .	. . .	. . .	. . .
Calf	*9*	*A2*	. . .	. . .	. T .	. . .	. . .	. . .	. . .	T G .	C . .	. . .	. . .	. C .	. . .	. . .	. . .
Calf	*11*	*A2*	. . .	. . .	. T .	. . .	. . .	. . .	. . .	T G .	C . .	. . .	. . .	. C .	. . .	. . .	. . .
Calf	*49*	*A1*	. . .	. . .	. . .	. . .	. . .	. . .	. . .	. . .	. . .	. . .	. . .	. . .	. . .	. . .	. . .
Calf	*52*	*A1*	. . .	. . .	. . .	. . .	. . .	. . .	. . .	. . .	. . .	. . .	. . .	. . .	. . .	. . .	. . .
Calf	*68*	*A1/A2*	. . .	. . .	. K .	. . .	. . .	. . .	. . .	Y S .	M . .	. . .	. . .	. M .	. . .	. . .	. . .
Calf	*113*	*A1*	. . .	. . .	. . .	. . .	. . .	. . .	. . .	. . .	. . .	. . .	. . .	. . .	. . .	. . .	. . .
Calf	*127*	*A1/A2*	. . .	. . .	. K .	. . .	. . .	. . .	. . .	Y S .	M . .	. . .	. . .	. M .	. . .	. . .	. . .

** Carvalho et al., 2011. Reference haplotype (X16702.1) identical to IgG2[a]. Variants identical to IgG2[b] allotype*
